# La médecine générale perçue par les étudiants de la faculté de médecine de Sousse (Tunisie)

**DOI:** 10.11604/pamj.2014.19.250.5006

**Published:** 2014-11-07

**Authors:** Chekib Zedini, Manel Limam, Mariem El Ghardallou, Menel Mallouli, Tarek Mestiri, Iheb Bougmiza, Thouraya Ajmi, Ali Mtiraoui

**Affiliations:** 1 Laboratoire de Recherche « LR12ES03 », Département de Médecine Familiale et Communautaire, Faculté de Médecine Ibn El Jazzar Sousse, Tunisie

**Keywords:** Médecine générale, enseignement médical, choix de spécialité, General medicine, medical training, specialty choice

## Abstract

**Introduction:**

Les étudiants en début du cursus suivent la même formation hospitalo-universitaire, cependant, ils sont peu nombreux à choisir délibérément la médecine générale comme discipline d'exercice médical. Notre objectif est de préciser auprès des étudiants en médecine, les facteurs qui déterminent le choix de la médecine générale comme carrière et leur vision de cette discipline.

**Méthodes:**

Etude descriptive transversale menée auprès d'un échantillon d’étudiants inscrits à la faculté de médecine de Sousse (Tunisie) pour l'année universitaire 2012-2013. Le recueil des données a été pratiqué par l'intermédiaire d'un questionnaire conçu pour les fins de ce travail. La saisie et l'analyse des données ont été effectuées par le logiciel SPSS 18.0.

**Résultats:**

Notre étude a porté sur 388 étudiants dont 69,5% étaient du genre féminin. L’âge moyen était de 22,1 ± 2,8 ans. Cent étudiants (25,8%) avaient un parent cadre de santé sans que cela n'ait une influence sur le choix de carrière. A l'entrée à la faculté, seulement 7,1% (n = 27) voulaient faire de la médecine générale leur carrière. Le changement de choix vers la médecine générale est statistiquement différent à l'entrée et à la sortie de la faculté chez les internes finissants.

**Conclusion:**

Afin d'attirer davantage les étudiants vers la médecine générale, il serait judicieux d'adopter des changements majeurs touchant l'enseignement théorique et pratique et de modifier les conditions de travail en fonction des attentes des générations futures.

## Introduction

Depuis plus d'un demi-siècle, le progrès de la technologie a participé à la création et au développement d'une médecine de spécialité dite « spécialités d'organes ». Ces dernières se sont développées dans la majorité du temps dans les centres hospitalo-universitaires où s'effectue la formation pratique des étudiants en médecine. Par ailleurs, bien que la médecine générale (MG) ou la médecine familiale (MF) soit devenue une spécialité clinique orientée vers les soins primaires et qu'elle occupe une place particulière dans le paysage de la santé et des systèmes de soins, elle n'est reconnue que depuis peu en tant que discipline scientifique et universitaire, avec un contenu spécifique de formation, de recherche de pratique clinique et des propres fondements scientifiques [1, 2].

En dépit de tous ces efforts, la médecine générale souffrait et souffre encore d'une mauvaise représentation de la part de la société et même des professionnels de la santé. Dans plusieurs pays, cette discipline n'attire pas assez de candidats [3] et en Tunisie, on constate le même phénomène. Avec une croissance plus rapide des médecins spécialistes dont le nombre a été multiplié par 6,9 contre 3,7 pour les généralistes entre 1981 et 2008 [4]. Les étudiants se trouvent ainsi confrontés à un dilemme lors du choix de leur carrière qui est soit de devenir médecin spécialiste, soit de faire de la médecine générale leur discipline d'exercice. En effet, plusieurs études ont démontré que les raisons qui font que les étudiants en médecine choisissent telle ou telle discipline comme carrière sont nombreuses et complexes [5–7].

C'est dans le but d'explorer cette multitude de facteurs qui influent le choix de la spécialité, que notre étude auprès des étudiants de la faculté de médecine de Sousse (Tunisie) s'inscrit. Elle a pour objectif de répondre aux questions suivantes: qui sont ces étudiants qui veulent devenir médecins généralistes? Quelle est leur vision sur la médecine générale? Quelles sont les facteurs qui pourraient influencer ou modifier cette vision au fur et à mesure que leur formation avance? Et enfin, leur formation académique et pratique les a-t-elle préparés convenablement à l'exercice de cette discipline?

## Méthodes

Il s'agit d'une étude descriptive de nature transversale qui a été menée auprès d'un échantillon d’étudiants inscrits à la faculté de médecine Ibn El Jazzar de Sousse (Tunisie) pour l'année universitaire 2012-2013. Les niveaux d’études des étudiants sont la première année (PCEM1), la cinquième année (DCEM3) et les internes de deuxième année (des étudiants ayant accomplis cinq ans d’études médicales et qui sont en période de stage pendant deux ans). La liste des étudiants des niveaux choisis par l'enquête a été récupérée auprès du service de scolarité de la faculté de médecine de Sousse. L’étude s'est déroulée sur une période de deux mois allant de début janvier à fin février 2013. Notre étude a été conduite de façon exhaustive auprès de tous les étudiants des niveaux prédéfinis.

Les étudiants du PCEM1 et du DCEM3 ont été contactés par passages répétitifs lors des enseignements dirigés en groupe selon l'organigramme affiché par la faculté. Les internes ont été contactés par passages répétitifs sur les lieux de stage. Pour les fins de ce travail, un questionnaire a été conçu, comportant 44 questions réparties en 5 rubriques: les caractéristiques sociodémographiques, la scolarité avant la médecine, leurs projets professionnels, l'influence de l'enseignement quant aux projets professionnels et leurs représentations de la médecine générale. Les variables quantitatives ont été décrites avec des moyennes ± leurs écarts-types et comparées par le test « t » de Student. Les variables qualitatives ont été décrites par des fréquences absolues et des pourcentages et comparées par le test du Chi deux lorsque les conditions de validité le permettaient, si non, le test exact de Fisher a été utilisé. Le seuil de signification a été fixé à 5%. La saisie et l'analyse des données ont été effectuées par le logiciel SPSS 18.0.

## Résultats

### Description de la population étudiée

Notre étude a porté sur 388 étudiants de la faculté de médecine de Sousse. Le Tableau 1 décrit la répartition selon le niveau d’étude et le taux de réponse. Notre échantillon avait une nette prédominance féminine (69,5%, n = 269). L’âge moyen des étudiants était de 22,1 ± 2,8 ans avec des extrêmes allant de 18 à 36 ans. Trois cent quarante quatre (90,8%) étudiants vivaient en milieu urbain. Trois cent cinquante neuf étudiants (93,7%) étaient célibataires. Quelle que soit leur motivation, leur sexe ou leur condition socioéconomique, les étudiants décident de suivre des études médicales de manière plutôt tardive. Ils sont près de la moitié (43,6%, n = 169) à se décider après le baccalauréat. Parmi ces derniers 10,1% (n = 17) ont choisi de faire de la MG leur carrière (à l'entrée à la faculté). Ce choix est statistiquement différent en fonction de la période (avant ou après le baccalauréat) (p = 0,035). Cent étudiants (25,8%) avaient un père ou une mère soit médecin (généraliste ou spécialiste) soit cadre paramédical. A l'entrée à la faculté, sept étudiants uniquement parmi ces derniers voulaient faire de la MG leur choix de carrière à l'entrée à la faculté. Alors que le reste des étudiants avaient choisi de devenir médecin spécialiste (p = 0,95).

**Tableau 1:** Répartition des étudiants selon le niveau d’étude et le taux de réponse

	Effectif	Pourcentage / effectif total (%)	Taux de réponse (%)
Etudiants de première année (PCEM 1)	190	49	67,4
Etudiants de cinquième année (DCEM 1)	136	35	71,2
Internes en deuxième année	62	16	37,1

### Facteurs influençant le choix des études médicales

Deux cent soixante neuf étudiants (70,1%) ont déclaré qu'ils avaient choisi de suivre des études médicales par choix personnel, 36,4% (n = 139) sous l'influence de la famille et 11% (n = 42) sous l'influence de séries télévisées. Tous niveaux confondus, deux cent quatre vingt dix sept étudiants (77,7%) étaient intéressés par la recherche ou la carrière universitaire. Trois cent cinquante quatre étudiants (92,9%) voulaient devenir médecins spécialistes, parmi eux cent trente neuf (39,3%) étaient indécis en ce qui concerne le choix de la spécialité. Les sept spécialités les plus choisies par les étudiants à l'entrée à la faculté sont représentés dans le Tableau 2. Seuls 27 étudiants (9 garçons et 18 filles) voulaient devenir médecins généralistes. Quant au rôle de l'enseignement dans le choix de spécialité, 143 étudiants (73,3%) du DCEM3 et des internes trouvaient que la médecine générale était insuffisamment abordée dans le programme de la faculté de médecine, alors que 161 (83,4%) pensaient que les études théoriques ne les ont pas préparés à l'exercice de la médecine dans la communauté. Parmi ces mêmes étudiants, 61,7% (n = 121) ont effectué un stage de MG. Ce dernier s'est déroulé dans un centre de santé de base (CSB) dans 92,4% des cas et dans un cabinet de libre pratique dans 7,6% des cas. Ce stage a été à l'origine d'une modification de la vision de la médecine générale dans 42% des cas et cette modification était plutôt positive dans 71,1% d'eux.

**Tableau 2:** Répartition des spécialités les plus choisies à l'entrée à la faculté

	Effectif	Pourcentage (%)
Indécis	139	36,5
Cardiologie	36	9,4
Médecine générale	27	7,1
Gynécologie	27	7,1
Pédiatrie	21	5,5
Chirurgie générale	20	5,2
Ophtalmologie	19	5
Psychiatrie	17	4,5

D'une façon générale, 50,5% des étudiants considéraient que le stage de MG n'offrait pas une image proche de la réalité, de même, que 68,2% des étudiants jugeaient leur formation pratique au deuxième cycle des études médicales comme non adaptée au futur médecin généraliste. Pour analyser l'impact de l'enseignement théorique (à la faculté) et pratique (aux stages) sur leur choix de spécialité, nous avons analysé séparément l'avis des étudiants du DCEM3 et des internes. Parmi les étudiants du DCEM3, 7,4% (n = 10) avaient été influencés par les cours magistraux (CM), 6,6% (n = 9) par l'enseignement dirigé (ED) et 63,2%(n = 86) par le stage d'externat. Quand aux internes, 4,9% (n = 3) avaient été influencés par les CM, 3,2% (n = 2) par les ED, 16,1% (n = 10) par le stage d'externat et 61,3% (n = 38) par le stage d'internat. Les enseignants ont participé à l'influence du choix de spécialité dans 66,2% des cas (n = 133), les médecins généralistes étaient source d'influence dans 21,1% (n = 28). Nous avons sélectionné les étudiants de DCEM3 et les internes afin de comparer leur choix de spécialité au début et à la fin du cursus (après au moins 5 années d’études médicales). Le changement de choix de la MG est statistiquement différent à l'entrée et à la sortie de la faculté chez les internes finissants et ce en faveur du choix à la sortie (p < 0,01) (Tableau 3).

**Tableau 3:** Distribution du choix de spécialité à l'entrée et après 5 ans d’études

	Choix de la MG à l'entrée		Choix de la MG à la Sortie		signification
	Oui	Non	Oui	Non	
5^ème^ année	9	127	10	126	NS
Internes finissant	5	57	15	47	P < 0,01
Total	14	184	25	173	NS

### Représentation de la médecine générale selon les étudiants

**Figure 1:**
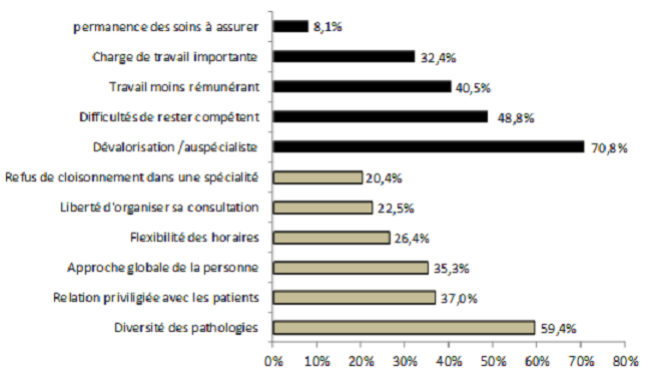
Les points positifs et négatifs de la médecine générale selon les étudiants

En ce qui concerne les caractéristiques positives et négatives de la médecine générale selon les étudiants, deux points importants apparaissent, à savoir une médecine générale caractérisée par une diversité des pathologies (59,4%) et une dévalorisation de la médecine générale (70,8%) par rapport à la médecine de spécialité (Figure 1).

## Discussion

L'originalité de notre étude réside dans le fait qu'elle élabore des données et des résultats qui proviennent d’étudiants de différents niveaux d’études (PCEM1, DCEM3 et internes finissants). Ceci permet de recueillir des informations concernant à la fois leur choix à l'entrée à la faculté et après au moins cinq années d’études ainsi que les changements d'avis tout au long du cursus quant au choix de la médecine générale comme carrière d'exercice médical. Le faible taux de participation est dû essentiellement au problème de disponibilité des internes surtout sur les terrains de stage et au désintérêt de certains étudiants. Dans notre étude, cent étudiants (25,8%) avaient un père ou une mère qui était soit médecin généraliste, soit spécialiste soit cadre paramédical mais il n'y avait pas de différence statistiquement significative entre le choix de la médecine générale comme carrière et la profession des parents. Cependant, le travail de D. Avery aux Etats-Unis d'Amérique en 2009 sous forme d'une revue de la littérature, portant sur les facteurs associés aux choix de la médecine générale comme carrière a trouvé que le fait d'avoir des parents qui ne font pas partie du domaine médical représente un facteur en faveur du choix de la médecine générale comme carrière [8].

A l'entrée à la faculté, la médecine générale était choisie par 27 étudiants (7,1%). Ce désintérêt pour la médecine générale a été retrouvé dans plusieurs études, par exemple en Grèce, il a été expliqué par le fait que la médecine générale est une discipline très généraliste (manque de spécialisation), suivie par la difficulté perçue à trouver un poste de carrière, la mauvaise acceptation des médecins généralistes par la communauté médicale et le faible prestige social [9]. Ce phénomène de désintérêt vis-à-vis de la médecine de famille a été constaté par d'autres auteurs et il s'amplifie au fil des années. En effet, en Angleterre plusieurs études ont montré une chute progressive et inquiétante de l′intérêt des étudiants en médecine pour la médecine générale, d′année en année [10, 11]. En ce qui concerne l'insatisfaction des étudiants vis-à-vis de l'enseignement théorique de la médecine générale au cours du cursus de formation, A.M Azzuro a relevé dans son étude faite dans 22 facultés en Espagne (2009/2010) que la formation en médecine familiale et communautaire durant les études médicales était considérée nécessaire puisque 92,2% des étudiants pensaient que la médecine familiale représentait une grande partie des compétences médicales [12]. En outre, une enquête du Comité National d′Evaluation de l′enseignement supérieur (CNE) a retrouvé que 71% des étudiants français estiment n'avoir jamais été sensibilisés à la médecine générale pendant leurs études [13].

Pour ce qui est des stages pratiques, une étude effectuée en Hollande (2008), a montré qu'après avoir effectué un stage en médecine générale, les préférences des étudiants pour cette discipline (tous sexes confondus) avaient nettement augmenté [14]. Une autre étude effectuée auprès des étudiants en médecine en Espagne a rapporté que 83,3% des répondants étaient convaincus de l'intérêt des stages dans les centres de soins de santé primaires [12]. De plus, notre étude a montré le rôle que jouent les enseignants non universitaires et particulièrement les médecins généralistes dans l'encadrement des étudiants sur les terrains de stages (externat et internat), surtout que ces derniers ont influencé la vision de certains étudiants de la médecine générale. En effet, l’étude de A.M Azzuro a trouvé que 73,6% des répondants approuvaient le fait que la médecine générale devrait être enseignée par des médecins de famille [12]. Pour étudier l’évolution du choix de la médecine générale comme carrière au fil du temps (début et fin des études), nous avons sélectionné les étudiants du DCEM3 et les internes à l'entrée à la faculté de médecine et après au moins cinq ans d’études. Nous avons ainsi constaté une augmentation statistiquement significative de 16,2% (8% Vs 24,2%) du nombre d'internes voulant faire de la médecine générale leur projet de carrière (p < 0,01). Nos résultats concordent avec l’étude de A.M Azzuro qui indiquait que la médecine familiale bénéficiait d'un intérêt croissant avec le temps, passant de 41,2% en première année à 48,7% en cinquième année (p < 0,001) [12]. De même H.Gill dans son étude effectuée en 2010 auprès des étudiants en médecine de la faculté d'Alberta au Canada, a constaté que l'intérêt des étudiants envers la médecine familiale grandissait au fur et à mesure que les étudiants progressaient dans leur parcours médical. Ainsi, la préférence pour la médecine familiale avait augmentée de 25% en première année à 36,1% en troisième année des études médicales [15].

Notre étude a fait apparaître quelques points importants, en ce qui concerne les caractéristiques positives et négatives de la médecine générale; Ces mêmes points ont été rapportés dans la littérature. En effet une étude qualitative par focus groupe faite auprès de seize étudiants en médecine Belges et douze étudiants Français (2009) a révélé que la médecine générale est considérée comme une médecine centrée sur le patient et cette relation privilégiée est perçue comme la pierre angulaire du métier et est la source principale de la motivation des jeunes médecins. Par ailleurs, certains étudiants sont moins enthousiastes et voient que travailler au quotidien près des gens et de leurs souffrances rend ce métier enrichissant mais parfois pénible. En outre, ils sentent que les médecins généralistes sont peu valorisés par la société, ainsi que le monde hospitalier et l’État malgré l'importance de la tache de ces derniers [3].

## Conclusion

Il s'avère que les étudiants en médecine continuent à préférer largement la médecine de spécialité à la médecine générale pour leur choix de carrière, ainsi pour attirer davantage les étudiants à la médecine générale, il serait judicieux: D'intégrer dans le cursus des études médicales à un stade précoce de la formation des périodes de stages qui devraient être plus attractifs, mieux pensés et permettent aux étudiants d'entrer en contact de façon précoce avec la communauté et surtout d’être plus sensibilisés aux vrais problèmes de santé de la communauté; De programmer dans le cursus des facultés de médecine l'enseignement de la discipline (MG) en mettant en exergue les caractéristiques de la médecine générale, les compétences fondamentales des médecins généralistes, l'impact des activités des médecins généralistes sur la communauté à court mais surtout à long terme (rôle de prévention et promotion de la santé); Modifier les conditions de travail des médecins généralistes en fonction des attentes des futures générations de médecins. Ainsi, il est clair que si l'on souhaitait réellement redorer le blason de la médecine générale, la volonté politique et les efforts des décideurs et des facultés de médecine ne devraient pas se limiter à une réforme (même si cette dernière permet d'amorcer certains changements) qui garde la MG comme une voie d’échec.
